# Why ophthalmology? Analysis of the motivating factors influencing the choice of ophthalmology as a career among different generations in Brazil

**DOI:** 10.6061/clinics/2019/e1101

**Published:** 2019-09-04

**Authors:** Gustavo Rosa Gameiro, Ana Letícia Fornazieri Darcie, Daniel Hazaki, Giovana Rosa Gameiro, Pedro Carlos Carricondo

**Affiliations:** I Faculdade de Medicina FMUSP, Universidade de Sao Paulo, Sao Paulo, SP, BR; II Departamento de Oftalmologia, Hospital das Clinicas HCFMUSP, Faculdade de Medicina, Universidade de Sao Paulo, Sao Paulo, SP, BR; III Faculdade de Medicina, Universidade Estadual de Londrina, Londrina, PR, BR

**Keywords:** Ophthalmology, Career Choice, Career Satisfaction, Motivation, Choice Behavior

## Abstract

**OBJECTIVES:**

The increasing demand for medical specialties with flexible working hours has been associated with the important role of quality of life as a determining factor when choosing a career in medicine, which might change the motivations for pursuing a career in ophthalmology. We aim to identify the main determinants of ophthalmology as a career choice as well as the reasons that motivated previous generations to follow this path.

**METHODS:**

Responses to self-administered online questionnaires were analyzed.

**RESULTS:**

A total of 225 responses were analyzed, including those of baby boomers (21), generation X (48), generation Y (131) and generation Z (25). Although the main reasons for choosing ophthalmology as a career are the same for all the generations in this study (flexible working hours, self-satisfaction from helping people improve their vision and the possibility of performing surgical procedures), some reasons for this career choice are more important to the younger generations (short-term results and short procedures), and some are more important to the older generations (the influence of an ophthalmologist in the family).

**CONCLUSION:**

The main reasons for choosing ophthalmology as a career are essentially the same over time. The differences in secondary motivations could be explained by generational differences.

## INTRODUCTION

In recent years, the search for professional areas with flexible working hours by new graduate physicians has attracted the attention of scholars worldwide, showing a shift in the profile of new physicians. A growing demand for positions in ophthalmology, dermatology, otorhinolaryngology, psychiatry and radiology has been reported 1-4.

The main motivations for choosing a medical specialty include the intellectual challenge, market perspectives and, recently, quality of life 5-9. The high importance given to work-life balance when choosing a medical career has been noted as being responsible for the increasing demand for certain specialties over other, more traditional areas such as internal medicine, pediatrics and general surgery 5-8,10-18.

Changes in motivation can be explained by generational differences, as members of each generation have their own attitudes, preferences and key characteristics. In spite of better dealing with differences and working better in a group, the current generation of medical students and residents, known as generation Y or millennials, is often described as being immediatist, desiring change, being individualistic and advocating for quality of life and leisure time 19-23. Their particular traits could influence their main motivations in their career choices.

Many studies have investigated the current reasons for choosing different medical specialties; however, only a few have focused on understanding the selection of less traditional specialties and the differences among generations. This study aims to characterize the main current determinants of the choice of ophthalmology as a specialty as well as the reasons that influenced previous generations to pursue this career.

## METHODS

### Participants

The sample was composed of Brazilian medical students participating in ophthalmology interest groups, ophthalmology residents in institutions accredited by the Brazilian Council of Ophthalmology and ophthalmologists in private practice. One thousand two hundred fifty-three subjects were contacted by email to answer a self-administered online questionnaire. Acceptance of the electronic consent form was necessary before proceeding to the questionnaire itself.

The participants were divided into generational groups according to their birth year (baby boomers: 1946-1964; generation X: 1965-1980; generation Y/millennials: 1981-1994; and generation Z: 1995-).

### Instruments

The instrument used in this study was an online self-administered questionnaire with objective questions regarding demographic information, reasons for choosing ophthalmology as a specialty, the presence of ophthalmologists in the family and the time of selection of the specialty. The questionnaire was sent via email by the Brazilian Council of Ophthalmology and the Brazilian Association of Ophthalmology Interest Groups.

**Figure 1:**
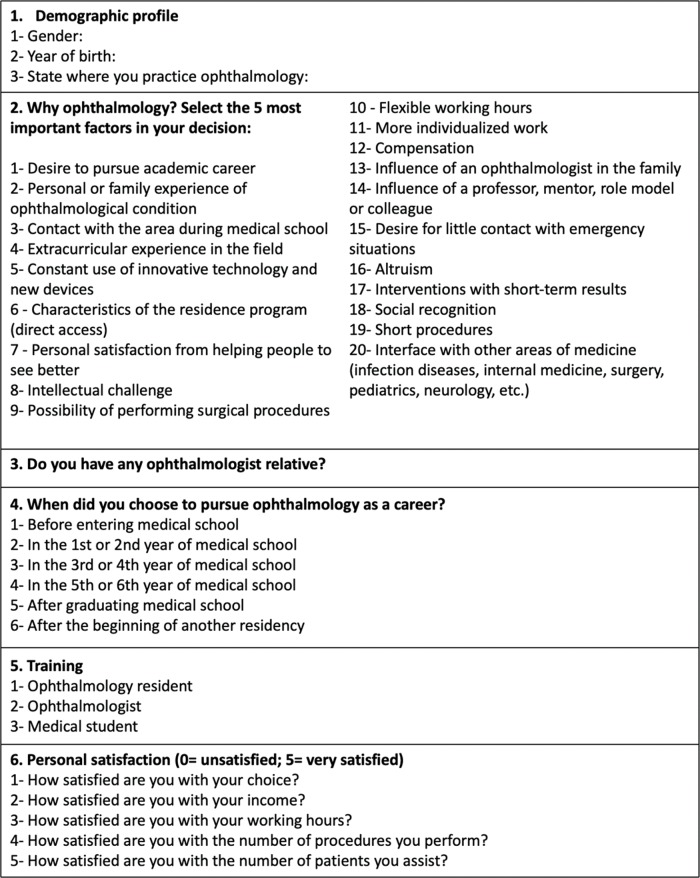
Questionnaire.

A sample of the administered questionnaire is shown in Figure 1.

### Data Analysis

Data analysis was performed with the aid of the computer program SPSS v.24.0 (IBM, Armonk, New York) using the appropriate tests for each variable, which are displayed after each table.

### Ethical Approval

Ethical approval for this study was obtained from the Research Ethics Committees (Comitês de Ética em Pesquisa - CEP) under protocol 2.622.328/2018.

## RESULTS

The response rate was 17.95%. A total of 225 responses were analyzed, which consisted of the responses from the baby boomers (n=21), generation X (n=48), generation Y (n=131) and generation Z (n=25) groups, as shown in Table 1. The participants included 114 ophthalmologists, 51 ophthalmology residents and 60 medical students (Table 1). There were no significant differences in the gender composition of the groups (Table 1).

The majority of the participants were from the Southeast Region (64%), followed by the Northeast Region (12%) and South Region (11%), as shown in Table 1.

When asked about having an ophthalmologist in the family, 70.7% of the participants in our study answered negatively (Table 1).

The main motivations behind each generation's choice of a career in ophthalmology as well as the significant differences among them are shown in Table 2.

The majority of the participants chose to follow ophthalmology as a career after being exposed to the area during medical school (77.7%), except for those who had a relative who was an ophthalmologist, who chose the career earlier, as seen in Table 3.

## DISCUSSION

### Old but still gold

In our study, we tried to identify whether younger generations aiming for a career in ophthalmology are influenced by different reasons than older generations in making their career choices, following the current trend of deciding on lifestyle-friendly residency programs 2-3.

According to previous studies 9,24-27, the main reasons for choosing ophthalmology as a career are the possibility of performing surgical procedures, flexibility, earning potential and intellectual stimulation.

**Figure 2:**
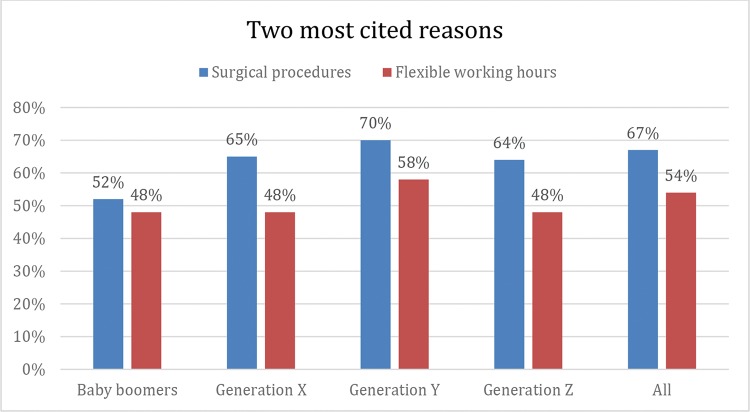
Percentage of the participants in each generation that indicated the possibility of surgical procedures and flexible working hours as important reasons for choosing a residency in ophthalmology. No significant differences were found among the groups (*p*-values of 0.415 and 0.519, respectively).

The strongest determining factors of career choices for all generation groups in this study were in accordance with the previous results in the literature, and there were no significant differences among the groups, showing that the main reasons for choosing ophthalmology may be essentially consistent over time (Figure 2).

### All things come to those who… want?

Although the main reasons for choosing a career in ophthalmology were the same for all groups, some reasons were significantly more important to the generation Y and generation Z physicians. This motivational trend can be explained by differences among generations.

**Figure 3:**
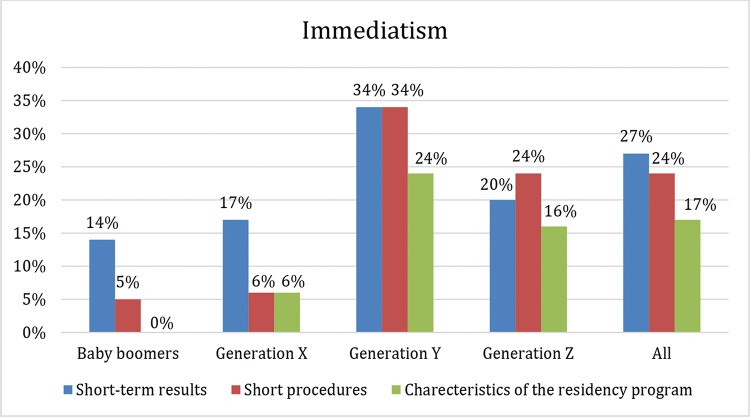
Percentage of the participants in each generation that chose short-term results, short procedures and the characteristics of the residency program (direct access) as important reasons for choosing ophthalmology. Generations Y and Z valued these reasons significantly more than the previous generations (*p*-values of 0.049, less than 0.0001 and 0.002, respectively).

Known for their impatience and immediatism, generation Y craves instant gratification, which may reflect the motivations that influence their career choices. In fact, direct access (no requirement to first complete an internal medicine residency) to ophthalmology residency programs and short-term results were significantly (*p*=0.002 and *p*=0.047) more important to the younger generations than to the older generations. Additionally, the short length of ophthalmic surgical procedures was statistically more likely to be identified by the generation Y and Z participants than by the participants from previous generations (*p*=0.000) (Figure 3).

### The apple does not fall far from the tree

**Figure 4:**
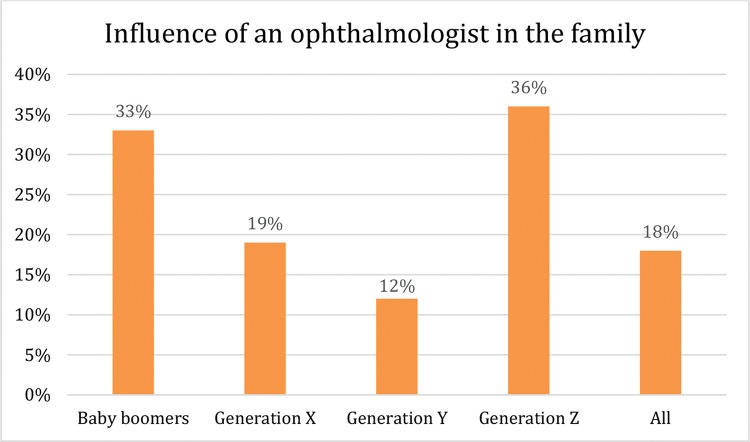
Percentage of the participants in each generation that indicated the influence of an ophthalmologist in the family as an important factor for choosing a career in ophthalmology. Generation Z participants and baby boomers were the most impacted by this circumstance, with 36% and 33% of the participants answering positively, respectively (*p*-value of 0.008).

The influence of an ophthalmologist in the family was noted as a determinant for the choice of ophthalmology by 33.3% of the baby boomers, 18.8% of the generation X participants, 12.2% of the millennials and 36% of the generation Z participants, with statistically significant differences among the groups (*p*-value of 0.008) (Figure 4).

Although having an ophthalmologist relative was not one of the most cited reasons, 34.8% of the participants who had an ophthalmologist in the family chose a career in ophthalmology before being exposed to the area during medical school; this finding differed from that for the other participants without an ophthalmologist in the family, as 83% of these participants had decided on a career in ophthalmology during the last years of their graduate studies (*p*-value of 0.003) (Table 3).

### Last and least

The least cited reasons among all generation groups for choosing ophthalmology as a career were altruism, the intellectual challenge and social recognition. This finding is inconsistent with the main determinants of the choice of medicine as a career that have been traditionally described in the literature 28-31. Whether future ophthalmologists will choose to attend medical school for different reasons remains to be investigated.

Pursuing an academic career was an important motivator for 28% of the generation Z participants and for 11% of the respondents overall (*p*=0.022). This result might arise from a selection bias since, at the time of the study, most of the participants in the generation Z group were involved in academic research activities linked to their participation in ophthalmology interest groups, which are organizations run by medical students interested in ophthalmology who organize and participate in extracurricular activities related to the field.

## CONCLUSIONS

The main reasons for choosing ophthalmology as a career differ from the determinants for deciding to be a physician. Whether a change in motivation occurs during medical school for those who decide on the ophthalmology career path remains to be investigated.

The differences in the motivations for deciding to become an ophthalmologist could be explained by generational differences. The immediatism of members of younger generations may impact their career choice determinants so that not only quality of life but also short-term results are highly valued. However, the main reasons for choosing ophthalmology are essentially consistent over time.

## AUTHOR CONTRIBUTIONS

Gameiro GR, Darcie ALF and Hazaki D contributed to the conception and design of the manuscript, literature review, manuscript drafting, critical revision and final approval of the version to be published. Gameiro RG contributed to the drafting of the final version of the manuscript, critical revision and final approval of the version to be published. Carricondo PC contributed to the conception and design of the manuscript, critical revision of the manuscript and final approval of the version to be published.

## Figures and Tables

**Table 1:** Demographic analysis of the participants.

Generation	Frequency	Percentage
Baby boomer	21	9.3
Generation X	48	21.3
Generation Y	131	58.2
Generation Z	25	11.1
Total	225	100
Level of training	Frequency	Percentage
Ophthalmologist	114	50.7
Ophthalmology resident	51	22.7
Medical student	60	26.7
Total	225	100
Gender	Frequency	Percentage
Male	121	53.8
Female	104	46.2
Total	225	100
Region	Frequency	Percentage
North	9	4.0
Midwest	19	8.4
Northeast	27	12.0
Southeast	145	64.4
South	25	11.1
Total	225	100
Ophthalmologist in the family	Frequency	Percentage
No	159	70.7
Yes	66	29.3
Total	225	100

**Table 2:** Main reasons for choosing a career in ophthalmology.

Reason	Baby boomer	Generation X	Generation Y	Generation Z	All	*p*-value
Surgical procedures	52%	65%	70%	64%	67%	0.415^a^
Flexible working hours	48%	48%	58%	48%	54%	0.519^a^
Personal satisfaction from helping people improve their vision	52%	40%	37%	44%	40%	0.598^a^
Compensation	24%	23%	38%	28%	32%	0.180^a^
Short-term results	14%	17%	34%	20%	27%	0.049*^a^
Influence of another person (mentor, professor, role model)	43%	29%	22%	20%	25%	0.182^a^
Technological innovations	29%	31%	24%	16%	25%	0.502^a^
Little contact with emergency situations	24%	19%	27%	20%	24%	0.622^a^
Short procedures	5%	6%	34%	24%	24%	0.000*^a^
Exposure to the area during medical school	19%	38%	19%	24%	24%	0.076^a^
Interface with other areas of medicine	29%	15%	16%	48%	20%	0.002*^a^
Personal experience of an ophthalmic condition	14%	31%	16%	16%	19%	0.144^b^
Influence of an ophthalmologist in the family	33%	19%	12%	36%	18%	0.008*^b^
Characteristics of the residency program	0%	6%	24%	16%	17%	0.002*^b^
More individualized work	19%	15%	16%	12%	16%	0.944^b^
Extracurricular experiences in the area	5%	21%	13%	20%	15%	0.260^b^
Academic career	0%	8%	10%	28%	11%	0.022*^b^
Intellectual challenge	5%	10%	6%	4%	7%	0.749^b^
Altruism	0%	4%	3%	8%	4%	0.479^b^
Social recognition	5%	0%	3%	0%	2%	0.483^b^

a -chi-square,

b - Fisher’s Exact test,

* - statistically significant.

**Table 3:** Time of career choice.

Chi-square = 0.003	No ophthalmologist in the family	Ophthalmologist in the family	Total
Before being exposed to ophthalmology	27	23	50
After being exposed to ophthalmology	132	43	175
Total	159	66	225

## References

[1] Gough HG (1975). Specialty preferences of physicians and medical students. J Med Educ.

[2] Schwartz RW, Jarecky RK, Strodel WE, Haley JV, Young B, Griffen WO (1989). Controllable lifestyle: a new factor in career choice by medical students. Acad Med.

[3] Lind DS, Cendan JC (2003). Two decades of student career choice at the University of Florida: increasingly a lifestyle decision. Am Surg.

[4] Kolcić I, Polasek O, Mihalj H, Gombac E, Kraljević V, Kraljević I (2005). Research involvement, specialty choice, and emigration preferences of final year medical students in Croatia. Croat Med J.

[5] Lieu TA, Schroeder SA, Altman DF (1989). Specialty choices at one medical school: recent trends and analysis of predictive factors. Acad Med.

[6] Burack JH, Irby DM, Carline JD, Ambrozy DM, Ellsbury KE, Stritter FT (1997). A study of medical students’ specialty-choice pathways: trying on possible selves. Acad Med.

[7] Alves MR, Naskashima AF (2003). Motivações e percepções de médicos residentes em relação è escolha da carreira em Oftalmologia. Rev Bras Oftalmol.

[8] Correia Lima de Souza L, Mendonça VR, Garcia GB, Brandão EC, Barral-Netto M (2015). Medical Specialty Choice and Related Factors of Brazilian Medical Students and Recent Doctors. PLoS One.

[9] Uemoto A, Kawamoto R, Abe M, Kusunoki T, Kohara K, Miki T (2015). The differences in speciality preferences and career determinant factors between first- and fifth-year medical school students. Nihon Ronen Igakkai Zasshi.

[10] Marschall JG, Karimuddin AA (2003). Decline in popularity of general surgery as a career choice in North America: review of postgraduate residency training selection in Canada, 1996-2001. World J Surg.

[11] Dorsey ER, Jarjoura D, Rutecki GW (2005). The influence of controllable lifestyle and sex on the specialty choices of graduating U.S. medical students, 1996-2003. Acad Med.

[12] Creed PA, Searle J, Rogers ME (2010). Medical specialty prestige and lifestyle preferences for medical students. Soc Sci Med.

[13] Parsa S, Aghazadeh A, Nejatisafa AA, Amini H, Mohammadi MR, Mostafazadeh B (2010). Freshmen versus interns’ specialty interests. Arch Iran Med.

[14] Takeda Y, Morio K, Snell L, Otaki J, Takahashi M, Kai I (2013). Characteristic profiles among students and junior doctors with specific career preferences. BMC Med Educ.

[15] Schwartz RW, Haley JV, Williams C, Jarecky RK, Strodel WE, Young B (1990). The controllable lifestyle factor and students’ attitudes about specialty selection. Acad Med.

[16] Newton DA, Grayson MS, Thompson LF (2005). The variable influence of lifestyle and income on medical students’ career specialty choices: data from two U.S. medical schools, 1998-2004. Acad Med.

[17] Tardiff K, Cella D, Seiferth C, Perry S (1986). Selection and change of specialties by medical school graduates. J Med Educ.

[18] Enoch L, Chibnall JT, Schindler DL, Slavin SJ (2013). Association of medical student burnout with residency specialty choice. Med Educ.

[19] Borges NJ, Manuel RS, Elam CL, Jones BJ (2006). Comparing millennial and generation X medical students at one medical school. Acad Med.

[20] Olson ME (2009). The “Millennials”: first year in practice. Nurs Outlook.

[21] Twenge JM (2009). Generational changes and their impact in the classroom: teaching Generation Me. Med Educ.

[22] DiLullo C, McGee P, Kriebel RM (2011). Demystifying the Millennial student: a reassessment in measures of character and engagement in professional education. Anat Sci Educ.

[23] Pingleton SK (2012). Millennial health care: change you can believe in. Chest.

[24] Noble J (2006). Factors influencing career choice in ophthalmology. Can J Ophthalmol.

[25] Noble J, Schendel S, Daniel S, Baerlocher MO (2007). Motivations and future trends: a survey of Canadian ophthalmology residents. Can J Ophthalmol.

[26] Abdulrahman M, Makki M, Shaaban S, Al Shamsi M, Venkatramana M, Sulaiman N (2016). Specialty preferences and motivating factors: A national survey on medical students from five uae medical schools. Educ Health.

[27] Lambert TW, Goldacre MJ, Bron AJ (2008). Career choices for ophthalmology made by newly qualified doctors in the United Kingdom, 1974-2005. BMC Ophthalmol.

[28] McManus IC, Livingston G, Katona C (2006). The attractions of medicine: the generic motivations of medical school applicants in relation to demography, personality and achievement. BMC Med Educ.

[29] Millan LR, Azevedo RS, Rossi E, De Marco OL, Millan MP, de Arruda PC (2005). What is behind a student’s choice for becoming a doctor?. Clinics.

[30] Wagoner NE, Bridwell SD (1989). High school students’ motivations for a career as a physician. Acad Med.

[31] Kleinert R, Fuchs C, Romotzky V, Knepper L, Wasilewski ML, Schröder W (2017). Generation Y and surgical residency - Passing the baton or the end of the world as we know it? Results from a survey among medical students in Germany. PLoS One.

